# The affective processing of loved familiar faces and names: Integrating fMRI and heart rate

**DOI:** 10.1371/journal.pone.0216057

**Published:** 2019-04-30

**Authors:** Jaime Vila, Cristina Morato, Ignacio Lucas, Pedro Guerra, Ana María Castro-Laguardia, María Antonieta Bobes

**Affiliations:** 1 Mind, Brain, and Behavior Research Center (CIMCYC), University of Granada, Granada, Spain; 2 Center of Neuroscience, La Habana, Cuba; Universidad Complutense Madrid, SPAIN

## Abstract

The neuroscientific study of love has been boosted by an extended corpus of research on face-identity recognition. However, few studies have compared the emotional mechanisms activated by loved faces and names and none have simultaneously examined fMRI and autonomic measures. The present study combined fMRI with the heart rate response when 21 participants (10 males) passively viewed the face or the written name of 4 loved people and 4 unknown people. The results showed accelerative patterns in heart rate, together with brain activations, which were significantly higher for loved people than for unknown people. Significant correlations were found between heart rate and brain activation in frontal areas, for faces, and in temporal areas, for names. The results are discussed in the context of previous studies using the same passive viewing procedure, highlighting the relevance of integrating peripheral and central measures in the scientific study of positive emotion and love.

## Introduction

Advances in the neuroscientific study of emotions have been slower for positive than for negative emotions. Part of this imbalance has been attributed to *negativity bias* [1], [2], [3], [4], which states that organisms are more prepared to attend to and defend themselves from situations that signal danger/punishment, than to attend to and approach situations that signal safety/reward. Negativity bias is evident in studies on approach-avoidance conflict both in animals [5] and humans [6]. The avoidance gradient -the increasing force with which organisms try to avoid a situation that is at the same time aversive and appetitive- is steeper than the approach gradient -the increasing force with which organisms try to approach that situation. The same bias is observed in the distribution of hundreds of visual and acoustic emotional stimuli from the IAPS (*International Affective Picture System*) and IADS (*International Affective Digitized Sounds*) when they are plotted in a two-dimensional space formed by the dimensions of valence and arousal [7], [8], [9]. The distribution of the pleasant pictures and sounds is less steep and more scattered than the distribution of the unpleasant pictures and sounds. The consequence is that there are fewer pleasant pictures and sounds that are highly activating than unpleasant ones, a situation that hinders the scientific study of positive emotions from a dimensional perspective when using the methodology of viewing/hearing standardized affective pictures and sounds [10].

The imbalance between positive and negative emotions is even more evident when emotional research is focused on discrete emotions [11]. Of the 5 emotions that most researchers in the field of emotion consider basic -anger, fear, disgust, sadness, and happiness-, only one corresponds to a positive emotion [12]. For years, research on the differentiation among discrete emotions included just a single positive emotion: happiness. Only recently has research interest on other positive emotions such as love, pride, amusement, or gratitude, started to grow [13], [14], [15], [16]. In the case of love, research interest has been boosted by increasing evidence of the fundamental role that social factors -in particular, social support and social cognition- play in brain functioning and health [17], [18]. Social support, defined as *the perception that one is loved*, *valued*, *and part of a social network*, has been known for decades to be associated with reduced morbidity and mortality rates [19], [20], [21]. This definition highlights the emotional component of social support that is essentially provided by family and friends and identified as love.

### Love through identity recognition: Faces and names

Social psychologists were the first to study romantic love, pointing out the presence of three basic components [22]: attachment, care-giving/receiving, and sexual attraction. The same components are assumed to be present in other types of love, such as maternal, filial, or fraternal love, except that sexual attraction is substituted with positive affect. From this perspective, love is better characterized as a motivational-emotional state that includes emotion, goal-directed motivation, and cognition [23]. Research on the emotional component of love has been facilitated by the extended corpus of research on face perception and identity recognition. Rapid access to information about people that we encounter is a prerequisite for effective social interactions [24]. The face is probably the stimulus that conveys more social and emotional information than any other stimulus. In Gobbini and Haxby’s model, recognizing the face of a person involves access to three types of information -visual familiarity, biographical information, and emotion- each one mediated by specific neural structures: a core system for visual familiarity (the inferior occipital/fusiform gyri and posterior superior temporal sulcus) and two extended systems for personal information (the anterior paracingulate, posterior temporal sulcus/temporo-parietal junction, anterior temporal cortex, and precuneus/posterior cingulate) and emotion (amygdala, insula, and striatum/reward system). A large number of studies have been published in the last two decades on face-identity recognition, including several reviews and meta-analyses, that support Gobbini & Haxby’s model [25], [26], [27], [28], [29], [30].

In a neuroimaging meta-analysis by Blank and colleagues [25] in healthy volunteers, studies on face-identity recognition also included the recognition of voices and names. Most of these studies focused on the cognitive processing of familiarity using tasks that required explicit recognition (i.e., providing the name of the person or judging whether a face/name was familiar or unfamiliar) or implicit recognition (i.e., gender-classification, one-back repetition, or inverted-target detection). However, a considerable number of those studies included a category of faces/names called personally familiar (faces/names of family members and friends) that involved stronger familiarity not only in terms of personal and biographical knowledge but also in terms of social attachment and emotion [24], [31], [32], [33] [34]. In addition, a subset of these studies, namely, those on face-identity recognition, focused on emotional processing and attachment by directly examining the faces of loved people, such as romantic partners, parents, or own children [34], [35], [36], [37], [38], [39], [40], [41].

### The passive picture-viewing paradigm

The majority of the studies on face-identity recognition and love limited the physiological investigation to the recording of central measures (mainly, event-related potentials and fMRI). Only a small group of studies have used Lang’s passive picture-viewing paradigm, a consolidated procedure that combines central and peripheral measures while participants view pleasant, neutral, and unpleasant pictures selected from the IAPS, to examine affective processing, [19], [42], [43], [44], [45], [46]. This paradigm is theoretically inserted into a dimensional model of emotion [7], where both positive and negative emotions are defined in terms of two basic affective dimensions -valence (pleasant versus unpleasant) and arousal (activated versus relaxed)- and conceptualized as action dispositions linked to two basic goal-directed motivational systems–appetitive motivation (positive emotions) and defensive motivation (negative emotions)-. The advantage of this paradigm is that it allows, by the combination of different physiological measures, the identification of affective processing and the differentiation between positive and negative affect. Highly arousing pleasant pictures (such as erotica and adventure pictures) are associated with a pattern of accelerative changes in heart rate, increases in zygomaticus major activity, decreases in corrugator supercilii activity, and inhibition of the startle reflex. The opposite pattern is associated with highly arousing unpleasant pictures (such as mutilated bodies and attack pictures). On the other hand, both highly arousing pleasant and unpleasant pictures are associated with larger skin conductance responses and larger event-related potentials (e.g., Late Positive Potential or LPP), indicating undifferentiated emotional arousal (either positive or negative). This paradigm has been applied by our group to the processing of loved familiar faces in four studies [19], [42], [43], [45]. In the first study [42], loved familiar faces included images of romantic partners, parents, siblings, and friends, and control faces included famous faces and unknown faces, in addition to baby faces from the IAPS. The second study [43] compared two loved faces: a face with higher familiarity–defined in terms of amount of time spent with the person- but lower emotionality–defined in terms of subjective report- (e.g., the father) and a face with lower familiarity but higher emotionality (e.g., the boyfriend). Control faces were the unknown faces of fathers and boyfriends of other participants. The third study [19] used three categories of faces: loved faces (romantic partner, father, mother and best friend), unknown-neutral faces (the faces of other participants), and unpleasant faces (mutilated faces from the IAPS). Finally, the fourth study [45, 46] compared loved familiar faces versus hated familiar faces, and unknown beautiful faces versus unknown ugly faces.

In these four studies, loved familiar faces repeatedly produced the expected pattern of physiological and subjective responses indicative of a genuine positive emotional response (heart rate acceleration, increased zygomaticus activity, decreased corrugator activity, startle inhibition, and reports of high positive valence and arousal) not confounded with familiarity, undifferentiated emotional arousal, or physical attractiveness. Notably, the pattern of the results was, in general, even stronger than the pattern obtained using the most arousing pleasant pictures of the IAPS (erotic pictures). Regarding brain activation, the results of the fourth study–the only one that included fMRI recordings on a separate session- revealed a pattern of brain activation in response to loved familiar faces (in contrast with unknown beautiful faces) with major clusters in areas involved in emotion and personal information: the medial orbito-frontal cortex, inferior frontal cortex, medial cingulate, and precuneus [45].

Only one study on name-identity recognition has applied the passive picture-viewing paradigm to the investigation of loved familiar names [47]. In this study, the same peripheral physiological measures used for faces were recorded while participants were viewing three categories of written names: loved familiar names (romantic partner, father, mother, and best friend), famous names (selected by each participant with instructions that the names did not evoke either positive or negative feelings), and unknown names (loved names of other participant). The results showed that loved names–compared to famous and unknown names- resulted in the expected pattern of physiological and subjective responses: heart rate acceleration, heightened zygomaticus and skin conductance activity, inhibition of corrugator activity, and reports of higher positive valence and arousal. Only one physiological measure (the startle reflex) behaved in the opposite direction showing startle potentiation instead of startle inhibition. This unexpected finding highlights a fundamental difference between the processing of faces and names. In the case of faces, the processing rests on perception, and the startle response behaves as in the IAPS picture-viewing paradigm, whereas in the case of names the processing rests on mental representation and imagery[48][49]. There is evidence that under these latter conditions the startle reflex behaves in the opposite direction than during the viewing of pleasant pictures, that is, startle potentiation instead of inhibition.

### The familiarity issue

The control of familiarity in studies on loved familiar faces and names has always been a major methodological problem. In this context, familiarity is defined as a form of explicit or declarative memory [24] [50]. This type of memory involves the ability to recollect events and factual knowledge about the person, which depends on many factors, including length of time spent with the person, number of previous encounters, duration of the relationship, or information accumulated about the person [38]. Attempts to control for familiarity include the use of acquaintances and famous people. However, familiarity of loved people will always exceed that of control faces and names because of the greater amount of time spent with them. In the studies reported above using the passive picture-viewing paradigm, familiarity was indirectly controlled by using faces and names of famous and unknown people [studies 42 and 47] and by comparing faces with different levels of familiarity and emotionality [studies 19, 43, and 45]. Participants in the latter set of studies were students who had lived in the family home with their parents until they were at least 18 years old. In contrast, their relationship with their romantic partner could not have exceeded a period of 6 years.

When loved familiar faces and names were compared with famous and unknown faces and names significant differences appeared in all physiological and subjective measures indicative of high positive affect, with no significant differences between famous and unknown faces and names as these both appeared emotionally neutral. Comparisons of loved familiar faces with different levels of emotionality and familiarity (romantic partner versus parent of same sex) also revealed differences indicative of higher positive affect for the less familiar one, although these results were limited to some physiological and subjective measures (zygomaticus, skin conductance, and arousal ratings). Finally, comparison of familiar faces with similar familiarity but opposite emotionality (positive versus negative affect) revealed differences in physiological and subjective responses indicative of positive/negative affect (zygomaticus, corrugator, and valence ratings) but no differences in the responses indicative of undifferentiated emotional arousal (skin conductance and arousal ratings).

### The present study

The present study focused on the emotional component of love and tested whether loved familiar faces and names share identical mechanisms of affective processing using an adaptation of Lang’s passive picture-viewing paradigm that included the simultaneous recording of fMRI and one autonomic measure: heart rate. We selected heart rate because in the context of this paradigm the phasic heart rate response is a valid index of emotional valence (positive versus negative). Pleasant pictures elicit an accelerative pattern of heart rate changes, whereas unpleasant pictures elicit a decelerative pattern of changes, compared to neutral pictures. Previous neuroimaging studies on loved familiar faces that included autonomic measures–almost always limited to skin conductance, an index of undifferentiated emotional arousal- performed the peripheral recordings in a separate session due to the difficulty of controlling electromagnetic interferences within the scanner. The present study partially overcame the interference problem by applying appropriate sensors and filters to the recording of the heart rate within the scanner. Although the problem was not completely eliminated, this methodology resulted in a sufficient number of participants with data that was free of electromagnetic artifacts. We also selected very close relatives as stimuli (tailored to each participant) to present only faces and names of very dear people (romantic partner, father, mother, and best friend), following the same criteria as in previous studies, to guarantee the elicitation of positive affect. Control stimuli were faces and names of unknown people (taken from other participants) to guarantee neutral affectivity. Thus, our design had two factors: Affectivity (loved versus neutral) and Picture Modality (faces versus names). Since the focus of the comparison was the two categories of loved stimuli (loved faces versus loved names), no control stimuli for familiarity were used. Regarding the fMRI data, we expected to find, for both faces and names, brain activation in the neural structures suggested by the literature as selectively activated by pleasant IAPS pictures [51] (mainly erotica and adventure pictures) and pictures of loved ones [24, 34, 36]: the medial prefrontal cortex, insula, anterior cingulate, and nuccleus accumbens. In addition, we expected to find brain activation in areas specifically involved in personal information, such as posterior cingulate and precuneus, for both loved faces and names [24]. Finally, we also expected to find positive covariations between the amplitude of the heart rate accelerative response to loved faces and names and brain activation.

## Materials and methods

### Participants

The sample consisted of 21 healthy volunteers (10 males) aged between 18 and 35 years (M = 21.7). They were all Caucasian and required to have a positive relationship with their parents, romantic partner, and best same-sex friend. All participants evaluated each one of those relationships, in advance and on the day of the fMRI session, as satisfactory or very satisfactory using a 5-point Likert scale (1 = very unsatisfactory and 5 = very satisfactory). They were also required to provide in advance of the experimental session a photograph of them following specific instructions (see below). None of the participants reported current physical or psychological problems, and none were under pharmacological treatment. They had normal or corrected-to-normal vision (compatible glasses for fMRI were provided if participants needed them). All participants provided written informed consent to the study protocol and received course credits for their participation. The Ethics Committee of the University of Granada approved the study.

An additional sample of 10 participants (2 males) was not included in the final sample of 21 participants due to electromagnetic artifacts in the heart rate data. However, the fMRI results of these 10 participants were used as fMRI localizer for correlation analysis and are reported in the supplementary material.

### Stimuli and experimental procedure

The study was entirely conducted inside the fMRI scanner, with the participant laying down and looking at a monitor using a mirror mounted to the head coil. Stimuli were presented using the software *Presentation* (Neurobehavioral Systems). Four different categories of stimuli were used: loved faces, unknown faces, loved names, and unknown names. Each category comprised four different stimuli: father, mother, partner, and subject’s best same-sex friend. For the unknown categories, the four stimuli of another participant, matched in age and sex, were used. Participants confirmed at the end of the experiment that they did not know any of the control stimuli. Each stimulus was presented 10 times during the session. In addition, a face of a baby was presented 20 times, as catch trials, interspersed throughout the session. The instructions given to the participants were to look at the stimulus presented on the screen and press a button with the index finger of their right hand when they detected the face of the baby. The objective of these catch trials was to maintain the subject’s attention across the session.

A total of 180 trials were presented. Each trial consisted of a fixation cross for 3–5 (mean = 4) seconds followed by one of the images, presented for 2 seconds. All trials were pseudorandomly ordered for each participant, controlling that no more than 2 stimuli of the same category (loved faces, unknown faces, loved names, unknown names, and baby face) were presented consecutively. The total duration of the experimental procedure was 18 minutes.

All stimuli were presented visually inside a plain gray circle in the center of the screen. Instructions to take the photographs required that the pictures should not be taken by the participants themselves and that all faces had a plain white background, showed part of the neck and hair, looked directly at the camera, and showed neutral emotional expression. Afterwards, the photographs were edited and matched for size, color (grayscale), brightness, and contrast. Names included first name and one surname. They were presented in black ink Times New Roman, centered inside the circle.

### Image acquisition

A Siemens 3T Tim Trio MR scanner system with a standard birdcage head coil for signal transmission/reception (MAGNETOM, Siemens, Healthcare, Germany) was used to acquire all images. BOLD-contrast-weighted echo-planar images (EPI) for functional scans consisted of 40, interleaved, axial slices of 2.2 mm thickness (with gap, 30%) that partially covered the brain from about -57.2 below to about 57.2 mm above the P-A plane. In-plane resolution was 3 x 3 mm, with the following parameters: FOV = 210 mm, matrix = 70 x 70; echo time (TE) = 23 ms; TR = 3 s with no time gap; flip angle = 90°. The first five volumes of each run were discarded to allow for T1 equilibration effects. Subsequently, a MPRAGE T1-weighted structural image (1 x 1 x 1 mm resolution) was acquired for coregistration and display of the functional data, with the following parameters: echo time (TE) = 2.52 ms, repetition time (TR) = 2250 ms, flip angle = 9°, and field of view (FOV) = 256 mm. This yielded 176 contiguous 1 mm thick slices in a sagittal orientation.

### Autonomic data acquisition and analysis

Heart rate was recorded with version 2 of the PPU (Pheripheral Pulse Unit) that the Siemens scanner has by default at a sampling rate of 50 Hz. The pulse sensor was located at the left index finger. HR was analyzed using MATLAB R2014a ECGLAB tool [52] and the KARDIA software [53]. In a first step, ECGLAB detected each heart period (inter-beat interval). In the second step, KARDIA transformed the inter-beat intervals into weighted averaged HR every half second during the four seconds following stimulus onset, using as baseline the weighted averaged HR one second before the stimulus. The HR value at 2.5 s, which coincides with the largest HR response amplitude, was used for the analysis of the fMRI covariation (see below).

Statistical analysis for HR was a 2 x 2 x 8 repeated measures ANOVA with the first factor being Affectivity (loved/neutral), the second factor being Modality (faces/names), and the third factor being Time (the 8 half-second bins following stimulus onset). The Greenhouse-Geisser epsilon correction was applied when needed to control for violation of sphericity in repeated measures designs. Results are reported with the F values, the original degrees of freedom, and the epsilon-corrected p score. Level of significance was set at p < 0.05.

### Functional data analysis

Functional data were analyzed using SPM8 and related toolboxes (Wellcome Department of Imaging Neuroscience; http://www.fil.ion.ucl.ac.uk/spm). Outlier functional scans and slices were repaired with the Artifact Repair Toolbox (Gabrieli Cognitive NeuroScience Lab; http://cibsr.stanford.edu/tools/ArtRepair/ArtRepair.htm). Afterwards, the images were slice-time corrected taking the middle slice as reference (using SPM8´s phase shift interpolation with the unwarp option) and then realigned to the first image in the session. The anatomical T1 image was coregistered with the EPI. Each participant’s T1 scan was bias corrected, then spatially normalized to MNI-space and segmented into gray matter (GM), white matter (WM), and cerebrospinal fluid using the unified procedure in SPM8. The parameters for normalization of the anatomical image were used to transform the functional scans to MNI space. Normalized images were spatially smoothed using an 8 mm Gaussian kernel. Data were high-pass filtered (128 s cut-off period).

A massive univariate general linear model (GLM) was applied. In the design matrix, the duration of each stimulus condition was convolved with the hemodynamic response function. The heart rate (measured at 2.5 s post-stimulus) was treated as parametric modulators of the stimulus events, in order to identify activity occurring at the time of processing that correlated with the subsequent change in heart rate. This produces a heart rate regressor for each stimulus condition. The six motion-correction parameters were included in the design matrix as nuisance regressors. A t-statistic was then obtained for each voxel for the contrast of interest in each subject. After this, second level random effects analysis were carried out over the contrasts: (a) loved faces > neutral faces, and (b) loved names > neutral names (for locating areas responding to affectivity) and over HR associate to each condition (testing for activity predicting the magnitude of heart rate response separately within each stimulus category). Threshold for second level analysis was set at p < 0.05, FWE corrected.

## Results

### Heart rate

**Fig 1:**
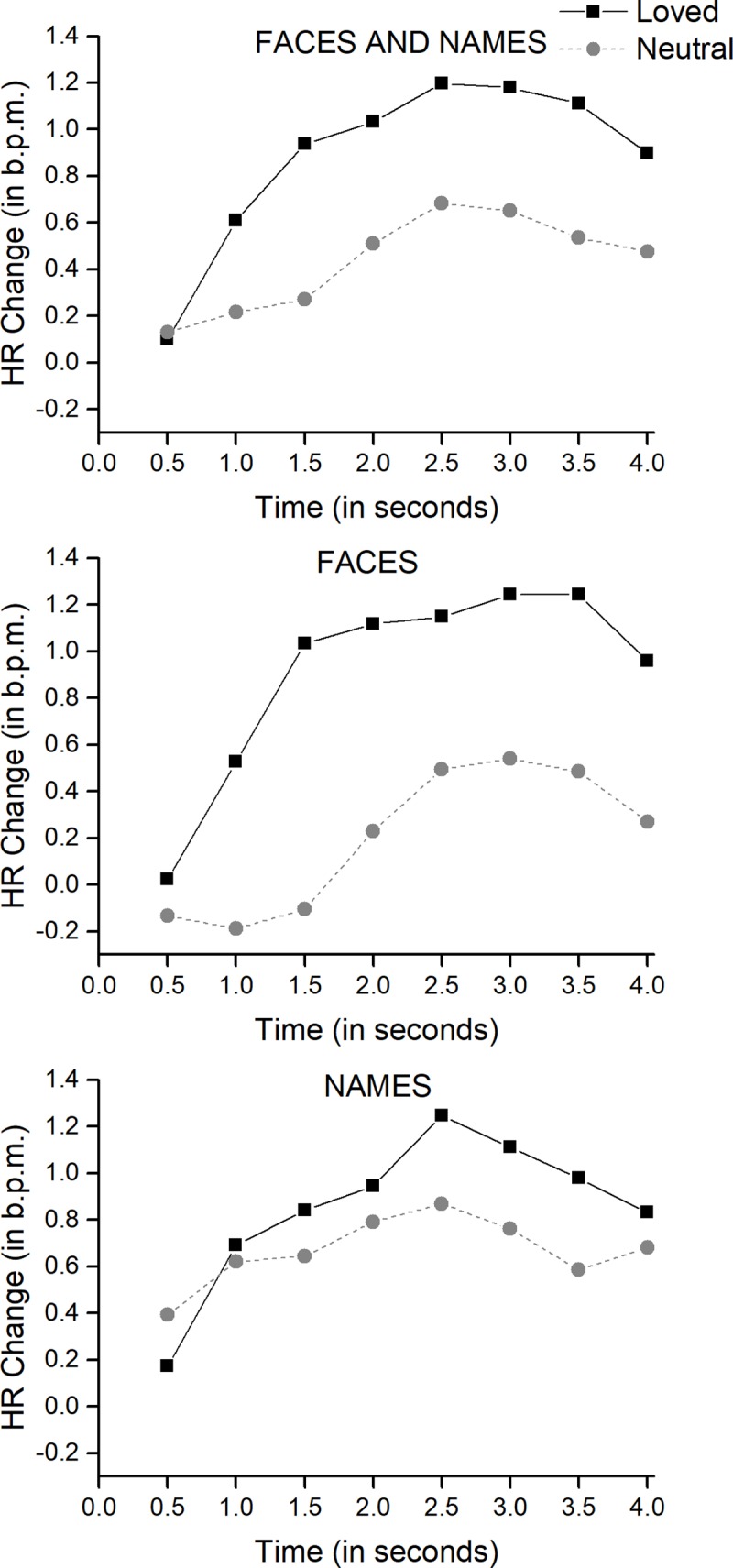
Heart Rate response to loved stimuli versus control stimuli. Top: averaged heart rate response to loved faces and names. Middle: averaged heart rate response to loved faces. Bottom: averaged heart rate response to loved names.

The 2 x 2 x 8 ANOVA yielded two significant main effects, Affectivity (F[1, 20] = 6.23, p < 0.02) and Time (F[7, 140] = 14.20, p < 0.0001). The Modality factor was close to significance (F[1, 20] = 3.81, p < 0.07). Fig 1 (top) plots the HR response in 8 half-second bins to loved stimuli (both faces and names) versus neutral stimuli (both faces and names). The response is in both conditions accelerative, reaching the peak amplitude at 2.5 s, and were significantly higher for loved stimuli than for neutral stimuli. Fig 1 (middle and bottom) plots the same data separately for faces and for names. Although not significant, this difference is larger for faces than for names.

### Functional imaging data

#### Affectivity effect

We tested a global Affectivity effect, by analyzing the contrast of loved (faces and names) > neutral (faces and names). No voxel survived the restricted threshold (FWE p < 0.05). Then we explore the specific Affectivity effect in each Modality. However, in order to explore the possible sites related to affective processing, the results of this contrast with a threshold of p < 0.01 (uncorrected) and cluster size > 50, are shown in Supplementary material (data in S1 Text, S2 Fig, and S1 Table).

#### Face affectivity effect

**Fig 2:**
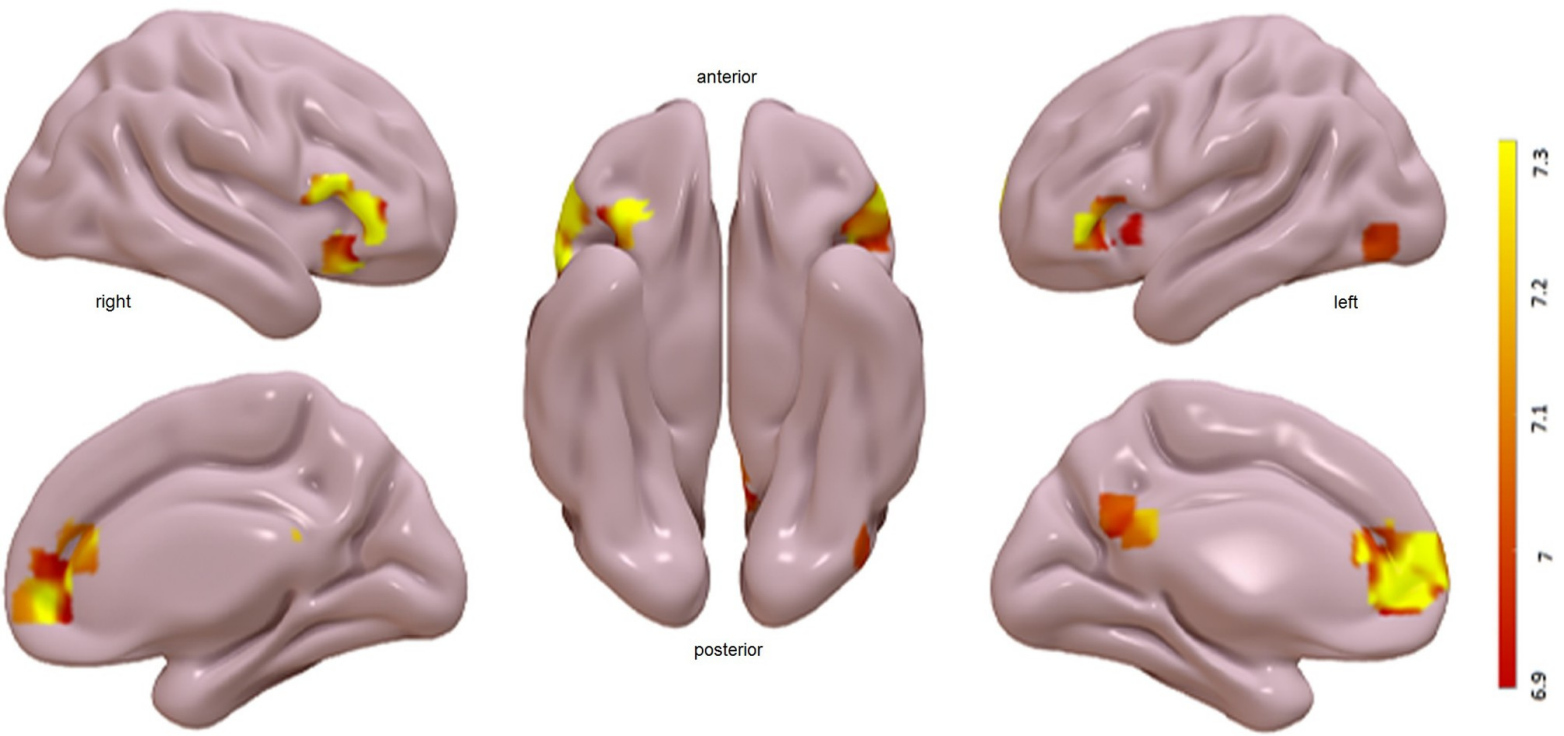
Face-Affectivity activations found on voxelwise analysis. Activation maps indicates regions where the response was higher for loved faces than for neutral faces. These activations are shown on an inflated brain depicting voxels surviving p < 0.05 (FWE corrected). Clusters of activations are observed in anterior cingulate (AC), medial orbito-frontal cortex (mOF), posterior cingulate (PC), and frontal inferior pars triangularis (FrinfTri).

Brain areas related to the processing of loved faces were located by analyzing the contrast of loved faces > neutral faces (see Fig 2 and Table 1). The responses to loved faces were larger than the responses to neutral faces in several regions, including anterior cingulate (AC), medial orbito-frontal cortex (mOF), posterior cingulate (PC), and frontal inferior pars triangularis (FrInfTri). Smaller clusters also appeared in other regions as shown in Table 1.

**Table 1:** Clusters of face-familiarity activations in a second level random-effects.

cluster	peak	Label	x,y,z {mm}		
p(FWE-corr)	p(FWE-corr)				
0.000	0.000	Frontal_Inf_Oper_R	58	20.0	6
	0.014	Frontal_Inf_Tri_R	52	30.0	2
0.000	0.002	Cingulum_Ant_L	-12	50.0	0
	0.003	Cingulum_Ant_L	-6	46.0	8
	0.006	Frontal_Sup_Medial_L	-8	54.0	12
0.000	0.010	Frontal_Med_Orb_R	10	52.0	-4
0.000	0.012	Frontal_Inf_Orb_R	50	32.0	-4
0.000	0.013	Cingulum_Ant_L	-2	40.0	16
0.000	0.016	Frontal_Inf_Tri_L	-44	24.0	0
0.012	0.019	Frontal_Inf_Orb_L	-46	32.0	-2
0.001	0.023	Frontal_Inf_Orb_R	28	26.0	-12
0.002	0.033	Precuneus_L	-2	-56.0	26
0.021	0.033	Cingulum_Post_L	-2	-46.0	22
0.021	0.039	Frontal_Inf_Orb_L	-30	32.0	-4
0.012	0.041	none	14	26.0	10
0.021	0.042	Cingulum_Post_L	-4	-48.0	20
0.012	0.046	Occipital_Inf_L	-46	-76.0	-8
0.021	0.046	Frontal_Sup_Medial_L	-10	66.0	10

Analysis for the localizar contrast (loved faces > neutral faces) p<0.05 (FWE corrected).

#### Name affectivity effect

Activations related to name Affectivity were studied through the contrast of loved names > neutral names. No voxel survived the restricted threshold (FWE p < 0.05). However, in order to explore the possible sites related to loved names processing, the results of this contrast with a threshold of p < 0.01 (uncorrected) and cluster size > 50, are shown in Supplementary material. In these conditions, activation, even though weaker, is observed in some areas also described for loved faces: frontal inferior pars triangularis and anterior cingulate. However, the most significant activations for this contrast appear in temporal superior and parietal areas (see data in S1 Text, S3 Fig, S2 Table).

#### Brain activity related to heart rate change across stimulus conditions

**Fig 3:**
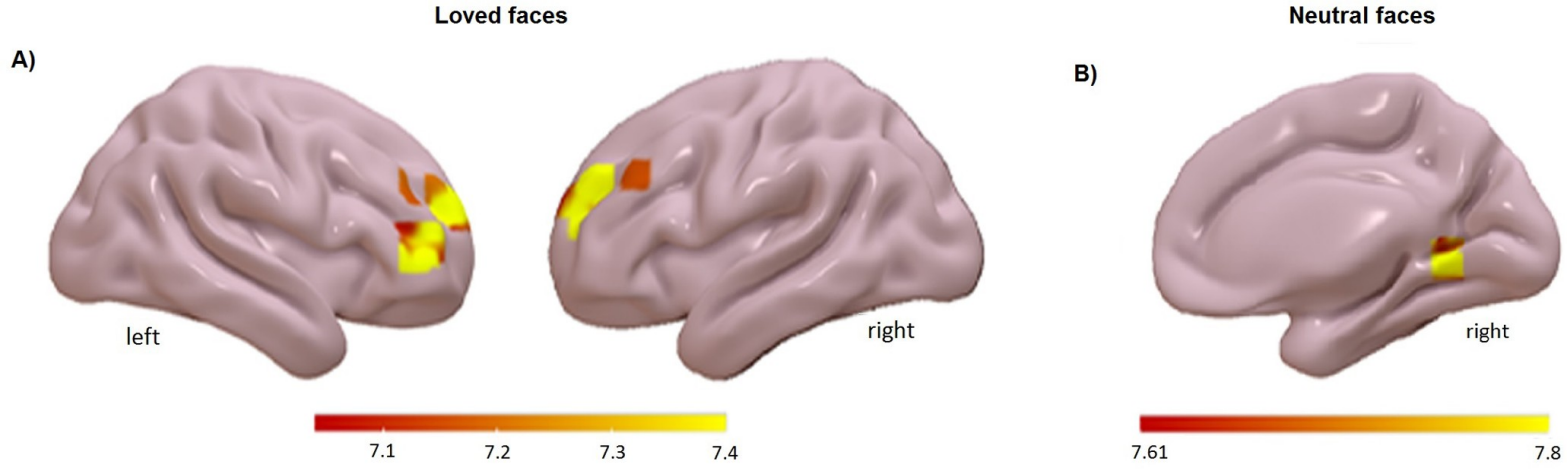
Correlational analysis of the evoked heart rate response to each face stimulus. The image shows the group effects in a second-level t-test for: A) loved faces and B) neutral faces, FWE corrected, p < 0.05 (only those clusters >10 are shown).

**Fig 4:**
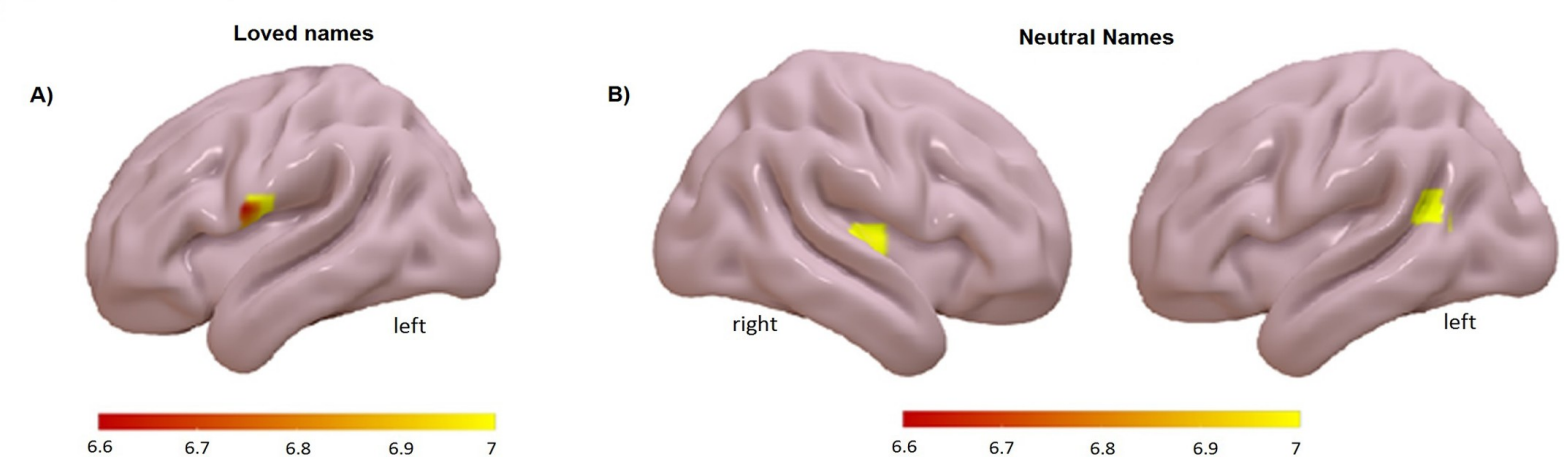
Correlational analysis of the evoked heart rate response to each name stimulus. The image shows the group effects in a second-level t-test for: A) loved names and B) neutral names. FWE corrected, p < 0.05 (only those clusters >10 are shown).

In order to determine which brain regions predict differences in magnitude of the heart rate response to faces and names we examined the effect of heart rate regressor associated to each stimulus condition. At first, such regions would show greater enhancement of activity to faces or names associated with larger evoked cardiac response. For each subject, we tested the effect of heart rate response to each stimulus condition and analyzed the group effects in a second-level t-test. Brain regions whose activity reflected evoked change in heart rate (at corrected significance) are shown in Fig 3 in the case of faces and Fig 4 in the case of names. For loved faces some clusters showed significant activations in the frontal area including the frontal inferior pars triangularis (FrInfTri R) (see Table 2). For neutral faces, few (and small) significant clusters were found, one located in FrInfTri R (see Table 2). For loved and neutral names, small clusters reach significance, located in precentral and temporal areas (see Table 2).

**Table 2:** Clusters showing significant correlations with the heart rate response.

Cluster p(FWE-corr)	Peak p (FWE-corr)	Label	x,y,z {mm}		
**Loved faces**					
0.000	0.000	Frontal_Mid_L	-26	48.0	28
0.000	0.000	Frontal_Sup_R	22	62.0	22
	0.017	Frontal_Mid_R	26	56.0	26
0.001	0.002	Frontal_Sup_L	-20	50.0	22
0.002	0.005	Frontal_Mid_R	44	46.0	4
0.004	0.012	Frontal_Sup_L	-24	60.0	22
0.000	0.013	Frontal_Mid_R	38	48.0	10
	0.016	Frontal_Mid_R	38	40.0	6
0.018	0.021	Frontal_Mid_L	-24	52.0	14
0.018	0.029	Frontal_Inf_Tri_R	46	42.0	2
0.018	0.032	Occipital_Sup_L	-22	-74.0	24
0.010	0.041	Frontal_Mid_R	36	42.0	30
0.018	0.045	Frontal_Mid_L	-40	32.0	32
**Neutral faces**					
0.021	0.000	none	-18	-32.0	54
0.000	0.004	Lingual_R	20	-54.0	-4
0.021	0.027	Frontal_Inf_Tri_R	46	36.0	10
0.012	0.027	Thalamus_L	-10	-22.0	4
0.012	0.031	Thalamus_R	6	-12.0	0
0.021	0.041	Lingual_R	14	-74.0	2
0.021	0.050	Lingual_R	18	-80.0	-12
**Loved names**					
0.017	0.031	Rolandic_Oper_L	-56	-6.0	12
**Neutral names**					
0.003	0.008	Insula_R	36	-16.0	4
0.009	0.015	Cingulum_Post_R	8	-44.0	28
0.013	0.017	Temporal_Mid_L	-44	-52.0	16
0.022	0.027	Temporal_Mid_L	48	-50.0	18

## Discussion

The present study showed heart rate responses that replicated, for the first time within an fMRI scanner, previous findings concerning loved familiar faces [19], [42], [43] and names [47]: larger increases in heart rate for loved faces and names than for neutral ones. Although not significant, there was also a tendency for the accelerative response to be larger for faces than names. Regarding the fMRI results, areas activated in response to loved compared to neutral stimuli were only significant for faces. However, activation for loved names seemed to be higher than activation for neutral names, but without reaching significance. This finding suggests that the activation strength of the visual perception of familiar faces surpasses the activation strength of the visual perception of their written names. In addition, the main areas activated by loved familiar faces were consistent with areas reported in the literature for personally familiar faces [25], [26], [27], [29]: the medial orbito-frontal cortex, frontal inferior pars triangularis, anterior cingulate, and posterior cingulate. Although the activation was weaker, some of these areas were also activated by loved names: the frontal inferior pars triangularis and anterior cingulate.

Regarding the relationship between the brain and autonomic responses, activity in a small group of brain regions predicted the magnitude of the cardiac responses to loved faces and names. For faces, relative increases in the heart rate response were accompanied by increased activity in frontal areas (frontal inferior pars triangularis), whereas for names, the cardiac changes were accompanied by increases in temporal areas, indicating that specific brain regions may support differential processing of faces and names. The regions related to heart rate responses to neutral stimuli are very small and located in different areas than those related to loved stimuli. It is interesting to note that the association between heart rate and brain responses related to loved names reached the restricted threshold set in this study, even when no significant results were obtained for name affectivity responses.

Although not the focus of the present paper (see Supplementary material), when loved familiar faces and names were combined, and a less restricted threshold was applied, two major clusters of activation were identified: one in the posterior cingulate and the other in the anterior cingulate and frontal superior medial areas. It would be very interesting to explore how these two systems (face and name) overlap with each other. However, to specifically investigate supramodal areas of activation for faces and names, different methods of analysis, such as multivoxel pattern analysis, would be necessary.

In general, these results suggest that loved familiar faces and names -although with different strengths- share some peripheral and central indices of affective processing. Below, we discuss the implications derived from these two types of measures and present an integrated account that highlights the contribution of using loved familiar faces and names to advance our knowledge on the neurophysiological mechanisms of positive emotions. First, we discuss the potential source of the observed differences between faces and names.

### Modality effect

The observed superiority of the visual perception of loved familiar faces over the visual perception of their written names may be explained by reference to two types of sources: studies showing differences between emotional stimuli of different sensory modalities and studies showing differences between perception and language processing. Studies comparing subjective, behavioral, and physiological responses to emotional stimuli in the visual and auditory modalities suggest no major differences when the stimuli represent naturally ocurring scenes or sounds such as the IAPS pleasant/unpleasant pictures and IADS pleasant/unpleasant sounds [54], [55]. However, when the emotional stimuli to be compared are facial expressions versus prosodic cues (voice tone), emotional recognition tends to be higher in the visual modality than in the auditory modality [56]. Similar superiority for the visual modality has been observed in the identity recognition of faces and voices [57]. Regarding research on perception and language processing, several studies have revealed an opposite pattern of responses concerning the modulation of the startle reflex by viewing emotional pictures and reading emotional words, which has critically been attributed to their different depth of processing [48], [49]. The same opposite pattern concerning the modulation of the startle reflex has been found when emotional pictures were loved familiar faces [19] and when emotional words were loved familiar names [47]. Unlike face processing, name processing seems to rest on mental representation, therefore, requiring deeper processing strategies.

### Heart rate

Our heart rate response to loved faces and names was a progressive accelerative response from the onset of the stimulus presentation, which reached maximum amplitude in approximately 2.5 seconds. Afterwards, the response tended to return to baseline. This pattern of response is similar to the heart rate response found in numerous studies using the passive picture-viewing paradigm when participants were viewing highly arousing pleasant pictures selected from the IAPS [7]. It is also similar to the heart rate response found in previous studies when participants were viewing loved faces and names [19], [42], [43], [47]. The major difference with respect to previous studies was in reference to the latency of the accelerative response. In previous studies, the accelerative response begins later, at approximately 1.5 seconds, and reached a maximum amplitude later, at approximately 3.5 seconds. This latency difference is explained by the different timing parameters of the stimulus presentations in the standard passive picture-viewing paradigm (approximately 6 s stimulus duration and 8 s inter stimulus interval duration) and in the present adaptation of the paradigm to meet the fMRI requirements (2 s stimulus duration and 3–5 s inter stimulus interval duration).

In the context of previous studies, there had been consistent evidence that this accelerative heart rate response is a valid indicator of positive affect. Negative affect is characterized by a decelerative heart rate response, whereas neutral affect produces no response or smaller responses. The covariation of this heart rate accelerative response with the brain activation observed in our study is necessarily explained by the complex connective network linking the heart and the brain including cortical and subcortical structures known to be involved in cognitive, emotional, and behavioral processes [58], [59], [60], [61], [62], [63]. Interestingly, some of these structures coincide with the cortical areas activated in our study by loved familiar faces and names: the medial orbito-frontal cortex and anterior cingulate.

### Central measures

The pattern of fMRI activation in response to loved familiar faces and names found in our study displays a set of overlapping areas that includes the anterior cingulate and inferior frontal areas (FrInfTri). The magnitude and extension of these activation levels were considerably larger for faces than for names. In addition, loved familiar faces showed strong activation levels in the medial orbito-frontal cortex and posterior cingulate. All these areas are known to be involved in both personal information and emotion. The posterior cingulate is part of the extended system in Gobbini and Haxby’s [24] model, being related to episodic memories. Although not included in the model, the medial orbito-frontal cortex and the anterior cingulate (but also the medial prefrontal cortex) are structures that recent studies have implicated in the processing of personally familiar faces and names [32], [33], [45], [64]. Furthermore, there is evidence that the medial orbito-frontal cortex is involved in emotional processing, a claim supported by data showing (a) suppression of the enhanced skin conductance responses by lesions to this area [63], (b) a significant correlation between BOLD signals and skin conductance amplitudes [65], (c) the role of the medial orbito-frontal cortex in the reward value of stimuli [66], and (d) its activation by emotional facial expressions [67] and pleasant imagery [51].

Regarding the anterior cingulate and the inferior frontal areas, they seem to play an important role in affective processing for both faces and names. The anterior cingulate and the inferior frontal gyrus have been implicated in a variety of cognitive and emotional processes including empathy [68] and subjective happiness [69]. The anterior cingulate was one of the areas activated in Bartels & Zeki’s studies [35] [36] where people viewed faces of their romantic partner or mothers viewed faces of their own children. These two areas showed intense and prolonged activation in Bobes et al.’s [33] study when participants viewed faces of acquaintances. Combined with the parallel increases in skin conductance responses to faces of acquaintances [70], our present finding of the anterior cingulate as one of the major structures in the processing of loved familiar faces and names -together with our finding of simultaneous increases in heart rate- supports the role that the anterior cingulate plays in the processing of positive emotional experiences.

### Contribution to the study of positive emotions: An integrated account

The passive picture-viewing paradigm, used in this and previous studies [19], [42], [43], [45], [47], contributes to advancing knowledge on the neurophysiological mechanisms of positive emotion, thus helping to reduce the gap between research on positive and negative emotions partially caused by the negative bias. The passive picture-viewing paradigm allows a large set of subjective and physiological measures that can be integrated to offer a tentative account of the psychophysiological mechanisms involved in the affective processing of loved people. Through this series of studies, the paradigm has provided (a) subjective indices of valence and arousal; (b) peripheral autonomic measures (heart rate and skin conductance); (c) peripheral somatic measures (zygomatic, corrugator, and orbicularis occuli muscles); (d) central electrophysiological measures (N2, P3, and LPP event-related potentials); and (d) central metabolic measures (fMRI).

From a subjective point of view, the faces of loved people induce strong feelings of positive affect (high valence and arousal). The peripheral physiology confirms the subjective data, showing a pattern of autonomic and somatic responses specifically indicative of positive emotion (increased heart rate and zygomatic muscle activity, together with decreased corrugator muscle activity and eye blink startle reflex) and emotional arousal (increased skin conductance). The central electrophysiological measures reveal three ERP components (N2, P3, and LPP) that have differentiated loved faces from all other face categories, providing insights on the temporal pattern of brain processes that underlie the recognition of loved familiar faces. N2 is the first ERP component modulated by loved faces and shows reduced amplitude. This reduced N2 is followed by increased P3 and LPP amplitudes. N2 has been interpreted in terms of action inhibition [71], [72], [38]. Within this interpretation, viewing the face of a loved person suppresses the action inhibition presumably to facilitate approach behaviors. The subsequently increased fronto-parietal P3 may indicate increased attentional allocation to the most cognitive-laden faces [73], whereas the subsequently increased LPP in parietal regions can be interpreted in terms of undifferentiated emotional arousal by the most affective-laden faces [7], [51].

Regarding the central metabolic measures, the fMRI data support Gobbini & Haxby’s model of face identity recognition revealing activation in the face core system and in the extended system for personal information (posterior cingulate and precuneus). In addition, loved faces showed other areas of activation in the anterior cingulate and inferior frontal areas, two areas that also showed a response to names, although the response was weaker. These two areas have been implicated in the modulation of the reward system, thus suggesting that the affective processing of loved familiar faces and names involves the modulation of specific areas (presumably via the amygdala and the mesolimbic-dopamine pathway) with the capacity to (a) inhibit defense reactions [19], (b) buffer fear learning and stress [74], [75], and (c) mediate the observed autonomic and somatic responses, thus contributing to the strong feelings of positive affect, attachment, and caring that characterize love. The functional significance of these complex mechanisms, as noted by Bartels and Zeki [36], is the maintenance and perpetuation of the species, guaranteeing the formation of firm bonds between individuals.

### Limitations

Our study had a loss of participants regarding the autonomic measure due to electromagnetic interferences in the scanner (10 participants). Although this limitation did not prevent statistically significant effects that were in line with prediction, methodological improvements to avoid electromagnetic interferences will be necessary in future studies. Our study did not dissociate the confounding effect of cognitive and emotional familiarity. Our control stimuli were unknown faces and names, which have less familiarity, in terms of both personal knowledge and emotion, than loved faces and names. Although not the aim of the present study, the dissociation between these two familiarity components will need to be further addressed in future studies.

## Conclusions

The present study combined fMRI and heart rate measurements and showed parallel changes in the autonomic nervous system and the functional brain when participants, using the passive picture-viewing paradigm, viewed loved familiar faces and names compared to unknown neutral faces and names. The autonomic measure revealed a pattern of heart rate increases, replicating previous findings concerning loved familiar faces and names. The fMRI measures showed higher strength for faces than names, but for both types of stimuli, there are some brain regions that predicted the magnitude of cardiac response. These regions included frontal and temporal structures for faces and names respectively, and the magnitude of brain and autonomic responses to love are correlated. This set of fMRI and heart rate data highlights the relevance of integrating central and peripheral measures to advance knowledge of the complex mechanisms underlying positive emotion and love.

## Supporting information

S1 Text Supplementary information. (DOCX)

S1 Fig Boolean mask used as restriction for correlation analysis between HR response and BOLD response. (TIF)

S2 Fig Affectivity activations found on voxelwise analysis. Activation maps indicates regions where the response was higher for loved (faces+names) than for neutral (faces+names). These activations are shown on an inflated brain depicting voxels surviving p < 0.01 (uncorrected). Clusters of activations are observed in superior temporal, inferior parietal, anterior cingulate, and inferior pars triangularis (FrIntTri).
(TIF)

S3 Fig Name-affectivity activations found on voxelwise analysis. Activation maps indicates regions where the response was higher for loved names than for neutral names. These activations are shown on an inflated brain depicting voxels surviving p < 0.01 (FEW uncorrected). Clusters of activations are observed in superior temporal, inferior parietal, anterior cingulate, and inferior pars triangularis (FrIntTri).
(TIF)

S1 Table Cluster of affectivity activations in second level random-effects. (DOCX)

S2 Table Clusters of name-affectivity activations in a second level random-effects. Analysis for the localizer contrast (loved names > neutral names), p<0.01 (FEW uncorrected).
(DOCX)
