# Supplementary material for: The affective processing of loved familiar faces and names: Integrating fMRI and heart rate
Source: PLoS One. 2019 Apr 30;14(4):e0216057. doi: 10.1371/journal.pone.0216057 (PMC6490893; doi:10.1371/journal.pone.0216057)
Supplement: S1 Text — (DOCX) [file pone.0216057.s001.docx]

**Supplementary material**

**THE AFFECTIVE PROCESSING OF LOVED FAMILIAR FACES AND NAMES: INTEGRATING fMRI AND AUTONOMIC MEASURES**

Jaime Vila, Cristina Morato, Ignacio Lucas, Pedro Guerra, Ana María Castro-Laguardia, & M. Antonieta Bobes

**Material and Methods: Boolean mask for correlation analysis.**

We performed an additional analysis in a sample of 10 healthy volunteers (2 males) aged between 18 and 35 ears (M = 21.4), not included in the sample described in Material and Methods. It was not possible to analyze the HR recorded from those individuals due to the presence of artifacts. We used this sample to localize the brain areas responding specifically to loved stimuli. We used the same procedure described in materials and methods, including the same paradigm, image acquisition parameters, and preprocessing pipeline. Regarding the analysis, in this case, a massive univariate general linear model (GLM) was applied, but only the six motion-correction parameters were included in the design matrix. A t-contrast was then performed for each voxel on each of the 10 individuals for loved (faces and names) > neutral (faces and names) conditions. Subsequently, a second level random effects analysis was carried out. Threshold was set at p < 0.01, uncorrected. The image containing all the voxels surviving the stablished significant level, was then converted in a boolean mask for using in the correlation analysis (see Materials and Methods from the main text).

The resulting random effects model for this contrast is shown in Fig 1 (S1 Fig).

**Figure 1.** Boolean mask used as restriction for correlation analysis between HR response and BOLD response.

**Results.**

***Global Affectivity effect***

For localizing those areas responding to global Affectivity we analyzed the contrast of loved (faces and names) > neutral (faces and names) for each subject with the less restricted threshold (p < 0.01 uncorrected and cluster size > 50). Results of the random effects model for this contrast showed two main clusters of activations: one in the posterior cingulate and other that included the anterior cingulate and frontal superior medial areas (see Fig 2 and Table 1: S2 Fig and S1 Table).

**Figure 2.** Affectivity activations found on voxelwise analysis. Activation maps indicates regions where the response was higher for loved (faces+names) than for neutral (faces+names). These activations are shown on an inflated brain depicting voxels surviving p < 0.01 (uncorrected). Clusters of activations are observed in superior temporal, inferior parietal, anterior cingulate and inferior pars triangularis (FrIntTri).

***Name Affectivity effect.***

Activations related to loved names were studied through the contrast of loved names > neutral names. No voxel survived the restricted threshold (FWE p < 0.05). However, in order to explore the possible sites related to familiar names processing, the results of this contrast with a threshold of p < 0.01 (uncorrected) and cluster size > 50 were obtained. Fig 3 shows these results. In these conditions, activation, even though weaker, is observed in some areas also described for loved faces: frontal inferior pars triangularis and anterior cingulate (see Fig 3 and Table 2: S3 Fig and S2 Table). However, the most significant activations for this contrast appear in temporal superior and parietal areas.

**Figure 3.** Name-affectivity activations found on voxelwise analysis. Activation maps indicates regions where the response was higher for loved names than for neutral names. These activations are shown on an inflated brain depicting voxels surviving p < 0.01 (FEW uncorrected). Clusters of activations are observed in superior temporal, inferior parietal, anterior cingulate and inferior pars triangularis (FrIntTri).
