# Supplementary figures and images for: The affective processing of loved familiar faces and names: Integrating fMRI and heart rate
Source: PLoS One. 2019 Apr 30;14(4):e0216057. doi: 10.1371/journal.pone.0216057 (PMC6490893; doi:10.1371/journal.pone.0216057)

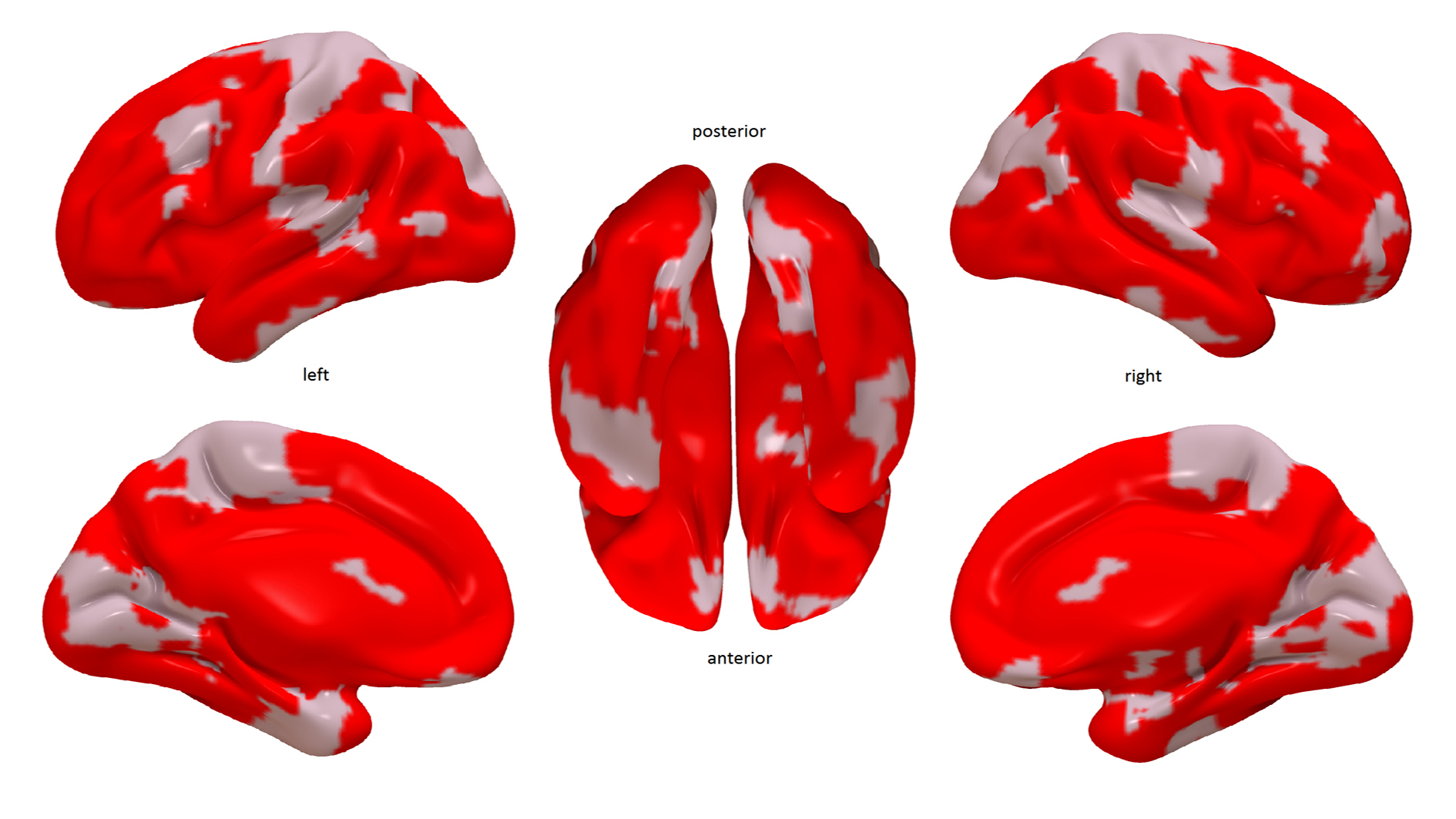

Supplement: S1 Fig — (TIF) [file pone.0216057.s002.tif]

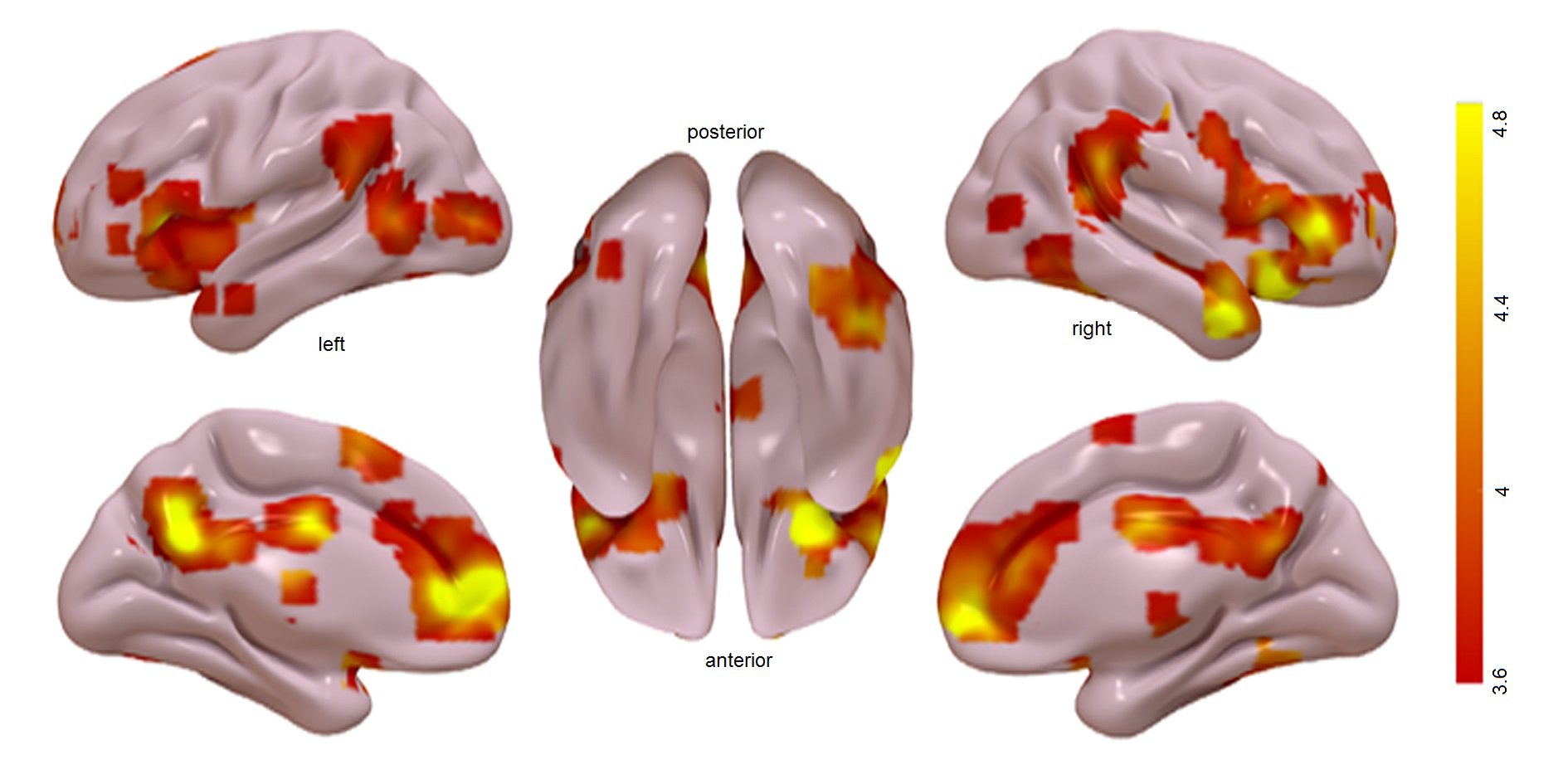

Supplement: S2 Fig — Activation maps indicates regions where the response was higher for loved (faces+names) than for neutral (faces+names). These activations are shown on an inflated brain depicting voxels surviving p < 0.01 (uncorrected). Clusters of activations are observed in superior temporal, inferior parietal, anterior cingulate, and inferior pars triangularis (FrIntTri). (TIF) [file pone.0216057.s003.tif]

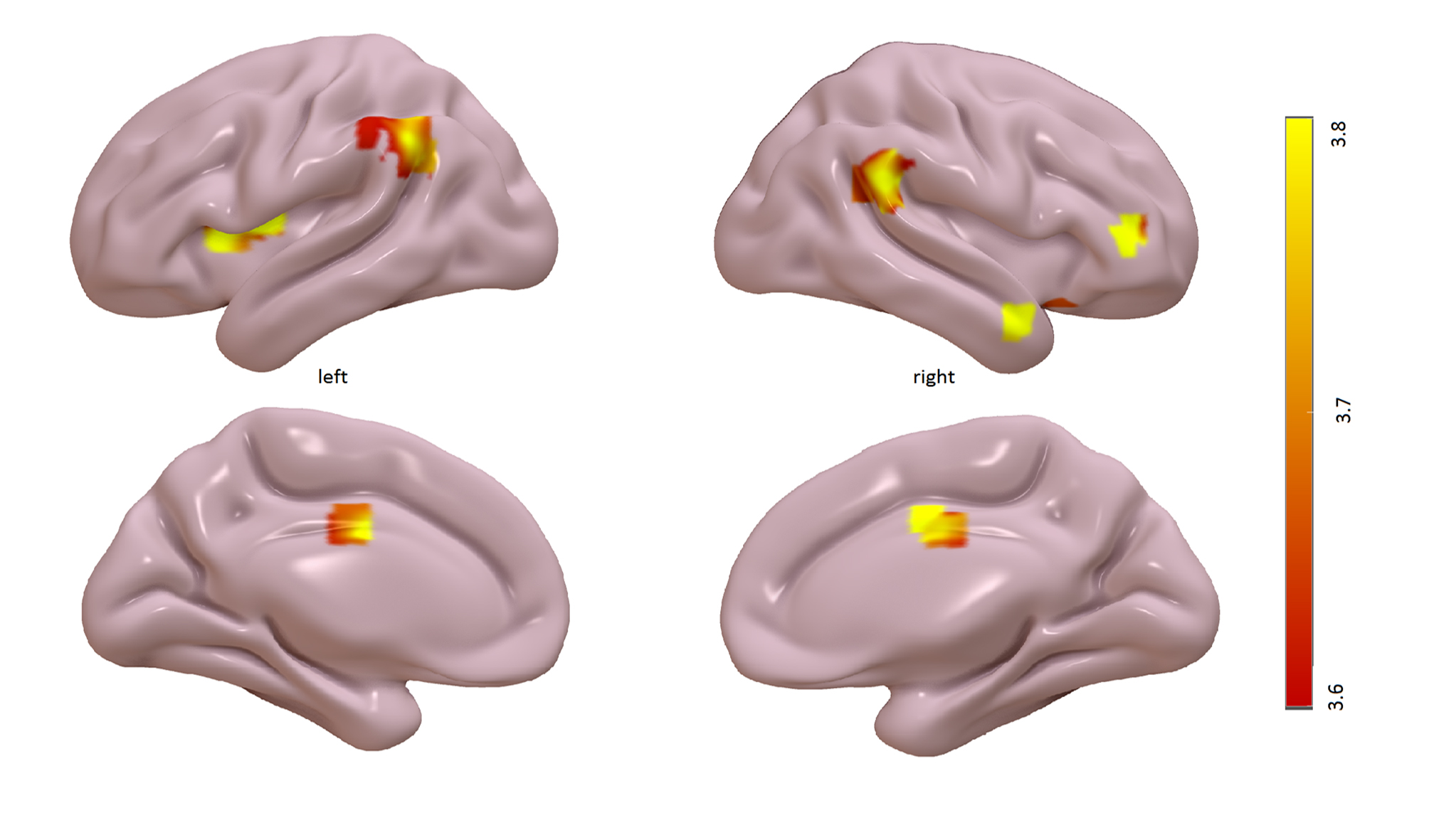

Supplement: S3 Fig — Activation maps indicates regions where the response was higher for loved names than for neutral names. These activations are shown on an inflated brain depicting voxels surviving p < 0.01 (FEW uncorrected). Clusters of activations are observed in superior temporal, inferior parietal, anterior cingulate, and inferior pars triangularis (FrIntTri). (TIF) [file pone.0216057.s004.tif]
