# Supplementary material for: The affective processing of loved familiar faces and names: Integrating fMRI and heart rate
Source: PLoS One. 2019 Apr 30;14(4):e0216057. doi: 10.1371/journal.pone.0216057 (PMC6490893; doi:10.1371/journal.pone.0216057)
Supplement: S1 Table — (DOCX) [file pone.0216057.s005.docx]

| **Table 1.** | | | | | |
| --- | --- | --- | --- | --- | --- |
| Clusters of affectivity activations in a second level random-effects. | | | | | |
| **Cluster** | **Peak p** | **Label** | **x,y,z{mm}** | | |
| **p(FWE-corr)** | **(FWE-corr)** |  |  |  |  |
| 0.007 | 0.002 | Insula_R | 28 | 20 | -14 |
| 0.009 | 0.000 | none | 16 | 22 | -10 |
| 0.012 | 0.000 | Caudate_R | 14 | 26 | -2 |
| 0.016 | 0.002 | Temporal_Mid_R | 54 | 4 | -28 |
| 0.006 | 0.000 | Temporal_Mid_R | 56 | 2 | -16 |
| 0.007 | 0.000 | Temporal_Mid_R | 48 | -2 | -20 |
| 0.000 | 0.006 | Frontal_Inf_Tri_R | 44 | 36 | 4 |
| 0.006 | 0.000 | Frontal_Inf_Oper_R | 58 | 20 | 10 |
| 0.006 | 0.000 | Frontal_Inf_Tri_R | 50 | 32 | 0 |
| 0.000 | 0.006 | Precuneus_L | -8 | -56 | 26 |
| 0.006 | 0.000 | Precuneus_L | -10 | -60 | 36 |
| 0.008 | 0.000 | Cingulum_Ant_R | -2 | -42 | 24 |
| 0.000 | 0.000 | Cingulum_Ant_L | 2 | 44 | 10 |
| 0.006 | 0.000 | Cingulum_Ant_L | -8 | 38 | 4 |
| 0.006 | 0.000 | Frontal_Inf_Tri_L | 0 | 52 | 12 |
| 0.000 | 0.000 | Thalamus_L | -48 | 24 | 4 |
| 0.006 | 0.000 | Pallidum_L | -18 | -12 | 4 |
| 0.007 | 0.000 | Temporal_Mid_R | -18 | 6 | 4 |
| 0.000 | 0.000 | Temporal_Sup_R | 44 | -46 | 16 |
| 0.006 | 0.000 | SupraMarginal_R | 66 | -48 | 20 |
| 0.007 | 0.000 | none | 62 | -44 | 26 |
| 0.016 | 0.000 | none | -2 | -10 | 30 |
| 0.407 | 0.000 | Temporal_Inf_R | 44 | -18 | -12 |
| 0.113 | 0.000 | Fusiform_R | 46 | -48 | -18 |
| 0.242 | 0.000 | none | 36 | -58 | -12 |
| 0.014 | 0.000 | Pallidum_R | 16 | 4 | 8 |
| 0.242 | 0.000 | Supp_Motor_Area_L | 24 | -4 | 4 |
| 0.147 | 0.000 | Occipital_Mid_L | -8 | 10 | 60 |
| 0.017 | 0.000 | Occipital_Mid_L | -32 | -86 | 4 |
| 0.020 | 0.000 | Temporal_Mid_L | -42 | -78 | 6 |
| 0.015 | 0.000 | Temporal_Mid_L | -52 | -58 | 6 |
| 0.019 | 0.000 | Temporal_Mid_L | -44 | -54 | 8 |
| 0.010 | 0.000 | SupraMarginal_L | -58 | -62 | 18 |
| 0.014 | 0.000 | Angular_L | -60 | -44 | 26 |
| 0.017 | 0.000 | Temporal_Sup_L | -58 | -52 | 34 |
