# Supplementary material for: The affective processing of loved familiar faces and names: Integrating fMRI and heart rate
Source: PLoS One. 2019 Apr 30;14(4):e0216057. doi: 10.1371/journal.pone.0216057 (PMC6490893; doi:10.1371/journal.pone.0216057)
Supplement: S2 Table — Analysis for the localizer contrast (loved names > neutral names), p<0.01 (FEW uncorrected). (DOCX) [file pone.0216057.s006.docx]

| **Table 2.** | | | | | |
| --- | --- | --- | --- | --- | --- |
| Clusters of name-affectivity activations in a second level random-effects. Analysis for | | | | | |
| the localizer contrast (loved names > neutral names), p<0.01 (uncorrected). | | | | | |
| **cluster** | **peak** | **Label** | **x,y,z {mm}** | | |
| **p(FWE-corr)** | **p(FWE-corr)** |  |  |  |  |
| 0.810 | 0.799 | none | -30 | 10.0 | 8 |
|  | 0.938 | none | -32 | -2.0 | 10 |
| 0.008 | 0.957 | Temporal_Sup_R | 64 | -42.0 | 24 |
|  | 1.000 | SupraMarginal_R | 60 | -44.0 | 32 |
|  | 1.000 | Temporal_Sup_R | 52 | -46.0 | 24 |
| 0.032 | 0.989 | Parietal_Inf_L | -54 | -50.0 | 38 |
|  | 1.000 | SupraMarginal_L | -50 | -44.0 | 32 |
|  | 1.000 | Parietal_Inf_L | -58 | -38.0 | 40 |
| 0.990 | 0.990 | Frontal_Mid_R | 36 | 40.0 | 6 |
|  | 1.000 | Frontal_Inf_Tri_R | 44 | 36.0 | 2 |
| 0.832 | 1.000 | Cingulum_Mid_R | 0 | -10.0 | 32 |
|  | 1.000 | Cingulum_Mid_R | 8 | -4.0 | 36 |
| 0.996 | 1.000 | none | -38 | -52.0 | 8 |
|  | 1.000 | Temporal_Mid_L | -48 | -64.0 | 6 |
|  | 1.000 | Temporal_Mid_L | -54 | -60.0 | -2 |
| 1.000 | 1.000 | none | -22 | 42.0 | 20 |
|  | 1.000 | Cingulum_Ant_L | -14 | 36.0 | 26 |
